# The complete mitochondrial genome of *Pseudopleuronectes herzensteini*

**DOI:** 10.1080/23802359.2022.2095234

**Published:** 2022-07-18

**Authors:** Jun Young Chae, JinHo Kim, Tae-Wook Kang, Dong-Gyun Kim, Hyung-Ho Lee, Moo-Sang Kim

**Affiliations:** a Department of Bioinformatics, MOAGEN, Daejeon, South Korea; b Department of Biotechnology, Pukyong National University, Busan, South Korea; c Biotechnology Research Division, National Institute of Fisheries Science, Busan, South Korea

**Keywords:** Mitochondrial genome, *Pseudopleuronectes herzensteini*

## Abstract

*Pseudopleuronectes herzensteini* belonging to *Pleuronectiformes* (family *Pleuronectidae*) is important in the fishery industry. However, the molecular biology of this valuable fish has hardly been reported. Thus, here we report the complete mitochondrial genome of *P. herzensteini*. The mitochondrial DNA (mtDNA) of *P. herzensteini* is 16,719 bp long and contains 13 mitochondrial protein-coding genes (PCGs), 22 transfer RNA (tRNA) genes, two ribosomal RNA (rRNA) genes, and a putative control region between *tRNA-P* and *tRNA-F* distinguished by a single short noncoding region. Phylogenetic analysis using PCGs confirmed that this mtDNA sequence belongs to the family *Pleuronectidae*. This is the first study reporting the complete mitochondrial genome sequence of *P. herzensteini*.

Littlemouth flounder (*Pseudopleuronectes herzensteini*; Jordan & Snyder 1901) is an economically important cold-water fish, belonging to *Pleuronectiformes* (family *Pleuronectidae*). *P. herzensteini* inhabits in East Sea of Korea, Northwest Pacific, Yellow Sea, inland Sea of Japan, and Kuril Islands (FishBase 2021). In South Korea, the annual catch has increased since 1990s (KOSIS 2021). Previous reports on *P. herzensteini* have mainly focused on ecological characteristics (Takahashi et al. 1995; Han and Kim 1999; Lee et al. 2006; Shimoda et al. 2006; Kobayashi et al. 2015) and the studies on genetic information have scarcely been reported. Thus, here we focused on the complete mitochondrial DNA (mtDNA) sequence of *P. herzensteini* and identified gene distribution, gene sequences, transfer RNA (tRNA) information, and phylogenetic relationships. This is the first study reporting the complete mtDNA sequence of *P. herzensteini* including the control regions.

The specimen was provided by the National Institute of Fisheries Science (NIFS, Busan, South Korea) and deposited in the Fisheries Bio-resources storage of NIFS (voucher no. NFRDI-FI-TS-0055174: https://www.nifs.go.kr/frcenter/, Dr. Dong-Gyun Kim, combikola@korea.kr). The gDNA was isolated from fin tissue using the Bead™ Genomic DNA Prep Kit for Animal Tissue (Biofact, Daejeon, South Korea). The *cox1* gene was amplified by PCR using the fish universal primer set, FishF2, 5′-TCGACTAATCATAAAGATATCGGCAC-3′; FishR2, 5′-ACTTCAGGGTG-ACCGAAGAATCAGAA-3′ (Ward et al. 2005). The amplicons were sequenced by Macrogen (Seoul, South Korea) and the *cox1* sequence was compared using the Basic Local Alignment Search Tool (BLAST) of the National Center for Biotechnology Information (NCBI). A BlastN (Johnson et al. 2008) search of the *cox1* sequence showed 98.96% similarity. to *cox1* sequence (MH032527) of the *P. herzensteini*, supporting that our flounder is *P. herzensteini*.

Library preparation for next-generation sequencing (NGS) was performed with MGIEasy DNA library prep kit (MGI, Shenzhen, China). NGS was conducted on the MGISEQ-2000 (MGI) with 150 bp paired-end reads. The raw data were deposited in Sequence Read Archive (SRA) database (SRR18558256). The mitochondrial genome of *P. herzensteini* was recovered by direct mapping to the *P. yokohamae* mitogenome (NC_028014) using Geneious ver. 11.1.3 (Kearse et al. 2012). The mtDNA of *P. herzensteini* showed 16,719 bp long circular DNA, and the sequence has been registered in the GenBank database (ON127848).

This complete mtDNA sequence was annotated using MITOS WebServer (Bernt, Donath, et al. 2013) and manually corrected using SnapGene software ver. 5.3.2 (GSL Biotech LLC, snapgene.com; Zheng et al. 2016). It constituted 13 protein-coding genes (PCGs), 22 tRNA genes, two rRNA genes, and the putative control region like general metazoan mitogenome components (Bernt, Braband, et al. 2013b).

Except *nad6*, other PCGs were transcribed on the positive strand started with an ATG codon (*nad1*, *nad2*, *cox2*, *atp8*, *atp6*, *cox3*, *nad3*, *nad4l*, *nad4*, *nad5*, *nad6*, and *cob*) except *cox1* which started with a GTG codon. The *nad2*, *cox2*, *nad3*, *nad4*, and *cob* were terminated with the truncated codons T-. The remaining six PCGs were stopped with TAA codon except for *nad5* and *nad6* where TAG codon was present. The results showed 22 tRNA genes including two *tRNA-L* and two *tRNA-S*. Except for seven tRNA genes (*tRNA-A*, *tRNA-N*, *tRNA-C*, *tRNA-Y*, *tRNA-S2*, *tRNA-E*, and *tRNA-P*), other 15 tRNA genes were encoded on the positive strand. The D-arm loop structure in the *tRNA-C* and *tRNA-S1* was not observed; however, the rest tRNAs retained the standard cloverleaf structure in the predicted secondary structure.

Small *rRNA* with a 949 bp length was located in between *tRNA-F* and *tRNA-V*, whereas large *rRNA* was located in between *tRNA-V* and *tRNA-L2* and had a length of 1716 bp. The putative control region was 1016 bp long and located in between *tRNA-P* and *tRNA-F*. The gene order was accorded with *P. yokohamae* (Zheng et al. 2016).

Several nucleotide sequences of PCGs from other related species, divided by family level for comparison with *P. herzensteini,* were collected from NCBI and *Acipenseridae* was utilized as an out-group. The phylogenetic tree was constructed using the MEGA 11 software (Tamura et al. 2021). The *P. herzensteini* was classified with *P. yokohamae* belonging to *Pleuronectidae* (Figure 1). Furthermore, *P. herzensteini* was gathered with *Pleuronectidae* not *Paralichthyidae* and *Bothidae*. The results of the present study evidence that the flounder analyzed in this study belongs to *P. herzensteini*.

**Figure 1.**
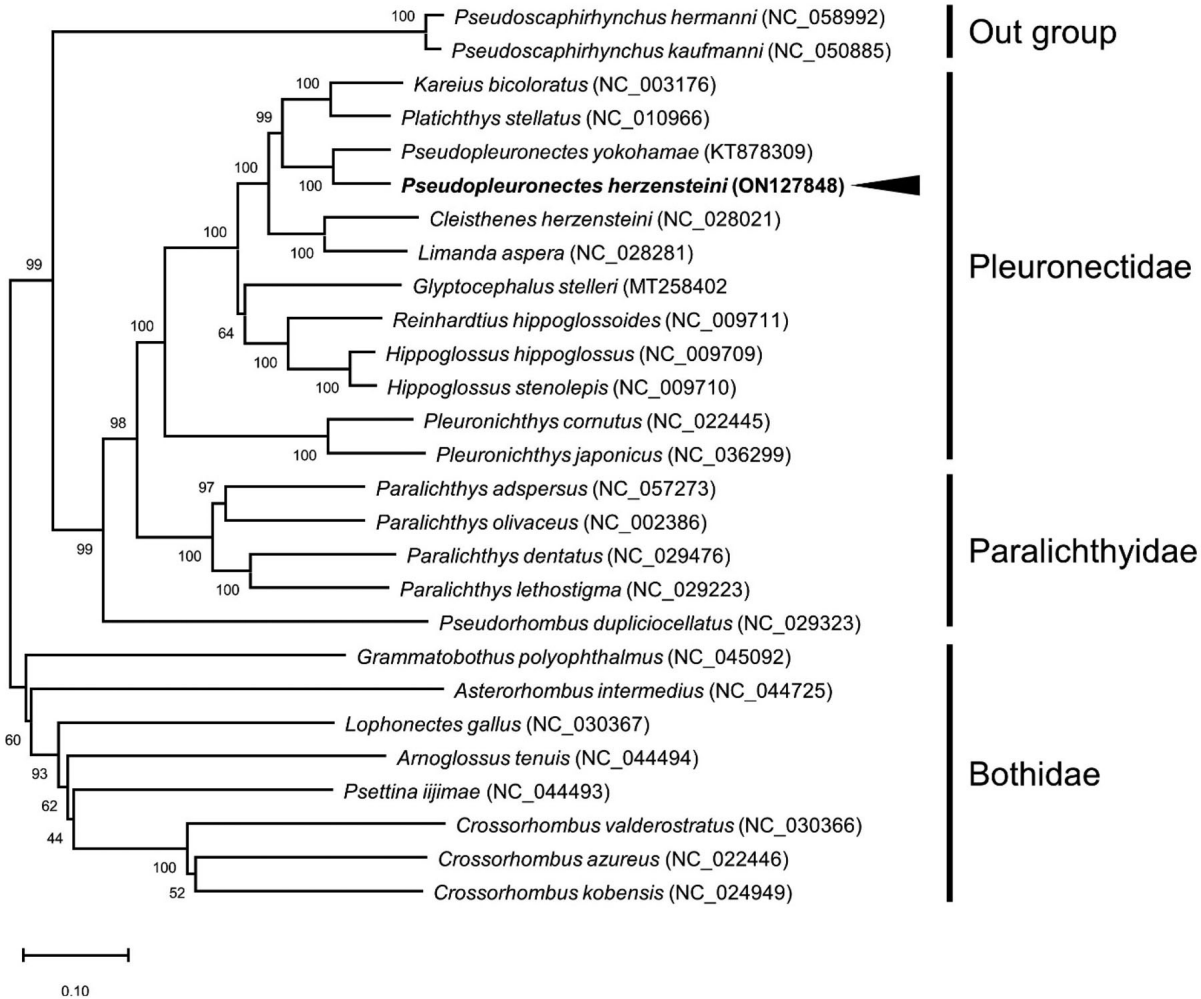
Phylogenetic tree of *Pseudopleuronectes herzensteini* and related species. Nucleotide sequences of PCGs of *Pseudopleuronectes herzensteini* obtained in this study and 26 related species were used to construct phylogenetic tree. Sequence data of other related species were obtained from NCBI. Neighbor-Joining method were conducted by repeating 10,000 times. *Bothidae*, *Pleuronectidae*, *Paralichthyidae*, and outgroup were indicated by vertical black bar. The black arrow indicates the *P*. *herzensteini* analyzed in this study.

## Data Availability

The genome sequence data that support the findings of this study are openly available in GenBank of NCBI at https://www.ncbi.nlm.nih.gov/ under the accession no. ON127848. The associated BioProject, SRA, and Bio-Sample numbers are PRJNA821835, SRR18558256, and SAMN27124384, respectively.
